# Study on the Influence of Measuring AE Sensor Type on the Effectiveness of OLTC Defect Classification

**DOI:** 10.3390/s20113095

**Published:** 2020-05-30

**Authors:** Daria Wotzka, Andrzej Cichoń

**Affiliations:** Faculty of Electrical Engineering Automatic Control and Informatics, Opole University of Technology, 45-758 Opole, Poland; a.cichon@po.edu.pl

**Keywords:** OLTC, AE sensor, acoustic emission, feature extraction, supervised classification, machine learning

## Abstract

The principal objective of this study is to improve the diagnostics of power transformers, which are the key element of supplying electricity to consumers. On Load Tap Changer (OLTC), which is the object of research, the results of which are presented in this article, is one of the most important elements of these devices. The applied diagnostic method is the acoustic emission (AE) method, which has the main advantage over others, that it is considered as a non-destructive testing method. At present, there are many measuring devices and sensors used in the AE method, there are also some international standards, according to which, measurements should be performed. In the presented work, AE signals were measured in laboratory conditions with various OLTC defects being simulated. Five types of sensors were used for the measurement. The recorded signals were analyzed in the time and frequency domain and using discrete wavelet transformation. Based on the results obtained, sets of indicators were determined, which were used as features for an autonomous classification of the type of defect. Several types of learning algorithms from the group of supervised machine learning were considered in the research. The performance of individual classifiers was determined by several quality evaluation measures. As a result of the analyses, the type and characteristics of the most optimal algorithm to be used in the process of classification of the OLTC fault type were indicated, depending on the type of sensor with which AE signals were recorded.

## 1. Introduction 

The reliability of the power system operation, to a great extent, depends on the proper operation of power transformers. These are devices constituting one of the main elements of the power transmission and distribution network. Their failures occur relatively rarely but result in huge costs. One of the most frequent causes of transformer failures is a faulty operation of on-load tap changers (OLTC). To ensure the continuity of the transformer’s electrical circuit and to maintain appropriate winding resistance parameters, the appropriate condition of OLTC contacts is essential. Due to the destructive action of the electric arc, the contacts are subject to wear processes. This phenomenon is particularly important in a power switch, where the switching process takes place at the flow of the transformer load current. Excessive wear of both fixed and movable contacts can lead to an increase in the contact resistance, which increases the temperature of the contact at the current flow and its further degeneration [1,2,3,4]. 

An important structural parameter of the contacts is their switching capacity, which depends on the way the contacts are made, the time of their closing and opening, and the medium in which the electric arc is extinguished. Due to the medium used to extinguish the electric arc and the method of breaking the electric circuit, OLTCs are divided into three groups: oily, with vacuum chambers, and thyristors. In this work, we consider OLTCs operating in electro-insulating oil.

There are many methods to determine the technical condition of power switches, which are based on vibration measurement, arcing or motor current/voltage signal analysis, dynamic resistance measurement, and acoustic emission (AE) signal measurement [2,5,6,7]. In [8], the authors investigated the possibility of using fractal analysis for damage detection in OLTC, while, in [9], a description of the dynamic resistance measurement (DRM) DV test method is given. In [10], a joint vibration and arcing measurement were applied for interpretation of events occurring during OLTC operation.

AE signals are often and successfully used to detect damage and assess the technical condition of equipment in the industry [11]. The advantage of the AE method is the possibility to carry out the technical condition tests of equipment or materials in a non-invasive way. The registered signals are processed in the time and frequency domain, with the use of various transformations, e.g., Fourier, discrete and continuous wavelet, Hilbert, and Gabor [2].

Today, scientists and engineers have relatively easy access to computers of high computing power. Moreover, there are numerous highly developed methods of artificial intelligence that are used in various fields. They are also successfully implemented for the diagnosis of electrical equipment. Classification tools, which are used when there exists a priori knowledge of the type of failure, can be applied in a process of supervised learning using a machine learning algorithm (MLA). If no knowledge of the type of defect is available, clustering methods can be applied using unsupervised learning. In the first case, genetic algorithms, hidden Markov models, chaos theory, group method of data handling (GMDH), fuzzy algorithms, and artificial neural networks (ANN) are usually used, in the second type, e.g., self-organizing maps (SOM) are applied. Classification or clustering is made based on a set of features, which are first obtained from registered measurement data. For example, the authors of the paper [12] proposed an expert system for the detection of various types of OLTC and circuit breakers. They proposed a feature extraction method based on a decomposition of acoustic signals in time and frequency domain. In their work, they used the reference database. However, this article does not give any detailed information about the algorithms specifically used, nor does it provide numerical values, based on which comparative analysis could be performed. In [13], the authors used an SOM neural network in the process of crack monitoring based on processed AE signals. In [14], authors apply supervised classification techniques and various feature extraction methods recognition of the aging state of a polyethylene-insulated cable for high voltage direct current (HVDC) usage. In [15] authors propose an efficient approach for classification of AE signals related to corrosion. They have applied random forests (RF) and k-nearest neighbor (KNN) algorithm. In [16], a hybrid method for the selection of features using the support vector machines (SVM) and KNN is proposed. Similar analyses in application to gear are described, e.g., in [17], where the authors obtained a maximum of 95.93% for testing accuracy when using psychoacoustic, and lower values for standard analyses. Similarly, not perfect accuracies were achieved in [18], where the authors apply the deep graph convolutional network for the diagnosis of faults of roller bearings. Rolling bearing fault diagnosis based on resonance-based sparse signal decomposition (RSSD) and waveform based on vibroacoustic signals are considered in [19], where the authors determine frequency spectrum and use these characteristic frequencies for fault diagnosis. The authors of [20] and [21] also use waveform transforms in their research works. In [21], a discrete wavelet transform and fast Fourier transform are applied for feature extraction and then a feed-forward with ANN was applied for recognizing the medium of the discharge source. In [22], authors applied ANN for the localization and identification of the AE source in various parts of the electric power transformer. In [23], the authors consider the influence of different feature sets on the results of SVM classification. They investigate rotating machinery using vibration signal for faults diagnosis. They analyze data in time, frequency and time–frequency domains, applying statistic features, empirical-mode decomposition, energy and Lempel–Ziv complexity features. They have achieved 78%-95% accuracy for the recognition of six types of failures. 

As shown above, there are many measurement and analytical possibilities to be used for diagnostics with the use of the AE method. However, there is a lack of knowledge on determining which algorithm of supervised machine learning is the most optimal to use depending on the type of measuring sensor. Therefore, our work attempts to answer this question. 

The main contribution of this work lies in the determination of the differences and selection of a set of optimal parameters (features) for the classification of technical condition of oily OLTC using the AE method, based on signals registered with various sensors. Furthermore, the novelty is in the analysis of the suitability of different sensors for OLTC fault detection and determination of the most suitable MLA for the particular sensor.

## 2. Materials and Methods

### 2.1. Measurement Setup 

The tests performed in the laboratory conditions were carried out using the OLTC model with a separate selector and power switch type VEL-110-27 from ELIN VEL-110. Inside the tank, there is a complete OLTC system consisting of a selector and a power switch located in the insulation sleeve. To limit the height of the system, the length of the tap selector has been reduced to six on/off switches. The tested OLTC model was placed in a tank filled with insulating oil. The system is equipped with a drive unit that enables the automatic switching of individual tapes. The change-over can be carried out between any two adjacent tapes. During the test, the entire tank was filled with insulating oil. In the presented measuring system, the tests were performed in two operating states: with nominal current flow and without load. The source used was three phase and supplied all three phases of the switch. The contacts in all three phases of the switch were connected to each other. Using a single-phase current forcing device, the 50 Hz AC flow was regulated from 0 to 250 A, in all phases symmetrically. Such a system guaranteed the possibility of generating an electric arc in all three phases of the power switch. The current value was generated at a low voltage of 50 V. The forcing device was located at a large distance from the measuring system, which limited the generation of possible interference from the transformer inductance. The contacts installed in the tested switch were the same as in the actual construction. Therefore, the results of the measurements of the discharges in the tested model of OLTC can be compared with the results obtained in the real object. In the laboratory conditions, comparative tests of particular types of measuring transducers were carried out. AE signals were measured with all transducers, which made it possible to perform a comparative analysis and to select the type of transducer for which changes in the AE signal structure generated by various defects in OLTC were most visible. The results of previous works, presenting the results of measurements and analyses carried out for the OLTC under consideration, can be found, e.g., in [24,25].

#### 2.1.1. Sensors Applied for Measurements 

The AE signal may be distorted and reverberated along with its propagation; therefore, the number and type of AE sensor, its installation, and placement must be carefully investigated [26,27]. In [28], authors investigate the influence of the distance between AE transmitter and receiver on the gathered analysis results. In this work, five types of acoustic-electric transducers are used to measure AE signals generated by OLTC:

- Broadband contact transmitter WD AH 17 (by Physical Acoustics Corporation) [29], marked as WD;
- Contact transducer D9241A (by Physical Acoustics Corporation) [30], marked as DS;
- Narrowband contact transducer R-15a (by Physical Acoustics Corporation) [31], marked as R15;
- Hydrophone 8103 (by Brüel & Kjær) [32], marked as MiG;
- Hydrophone TC4038 (by Reson) [33] marked as MiC.

The broadband transducer type WD AH 17 (WD) was attached to the outer wall of the ladle with a permanent magnet. The transducer used has a high sensitivity (55 dB ± 1.5 dB with V/ms^−1^) and a six-way bandwidth from 100 kHz to 1 MHz in the ± 10 dB range. This transmitter is equipped with a differential measuring system for the AE signals. The use of this system allows for eliminating interference signals, which appear under the influence of the electromagnetic field affecting the transmitter and measuring cable [34]. It is of particular importance while making measurements at the current flow during the OLTC switching process. This contact transducer is immune to the appearing interferences because of the differential system used to measure AE signals. Its frequency band allows for performing analysis of AE signals generated by OLTC in no-load condition, lower frequency bands as well as during current flow when components from higher frequency bands appear in AE signal.

The transducer type D9241A (DS) was also attached to the outer wall of the tank with a permanent magnet. This AE sensor is an epoxy sealed, enclosed device, which was developed for application in electrically noisy environments. Its ceramic face ensures proper insulation from the transformer tank. For noise reduction, the transducer is integrated with a differential BNC connector. Its typical operating frequency is in the range from 20 to 180 kHz range. Its sensitivity is around 82 dB V/(m/s).

The third type of contact transducer was the R15a—a narrow band device, which, similar to the other sensors, was mounted to the outer wall of the tank. The sensor cavity is built from a solid stainless steel rod, which makes it rugged and reliable. It also has a ceramic face for the improvement of the electrical isolation from the object under test. Its operating frequency is in the range from 50 to 400 kHz and its sensitivity of 63 dB with V/µbar. It enables application in a wide temperature range, from −65 to 175 °C.

The advantage of using a broadband contact transducer is the possibility of easy attachment to the tested object and analysis of the reactive controlled AE signals in a wide frequency band. However, the AE signal recorded with the use of contact transducers is distorted due to signal attenuation. It is created when the AE signal passes through barriers located inside the tested object [35]. When the AE signal is recorded in OLTC, the attenuation is caused by the necessity to pass the measured signal through the isolation sleeve, thick oil layer, and metal tank walls. 

For the comparison of AE signals occurring inside the tank with the signals recorded on its external wall, another measuring system using a TC4038 (MiC) hydrophone was used. The hydrophone was immersed in insulating oil between the wall of the ladle and the examined power switch. The hydrophone used has a wide bandwidth of 10 to 800 kHz within ± 5 dB and a high sensitivity of 228 dB ± 3 dB with V/µPa. During measurements, 30 dB amplification was used. The cut-off frequency of the high pass filter was 10 kHz, while the low pass filter was 1 MHz. 

For comparison purposes, the second hydrophone of type 8103 (MiG) was also applied. It enables AE signal registration within a wide frequency range, from 0.1 to 180 kHz, where it has a flat frequency response and with a sensitivity of 30 μV/Pa. This sensor is equipped with low noise, double-shielded integral cable, which enables appropriate electromagnetic shielding. The operating temperature is in the range from −40 to +80 °C. This type of sensor has a high level of corrosion resistivity; therefore, it may be applied in transformer oil. The use of the hydrophone allows for the precise analysis of time–frequency structures. This kind of transducer is, however, very sensitive to electromagnetic interferences arising from the flow of current through OLTC contacts. This makes the interpretation of the recorded signals much more difficult. The use of the hydrophone is mainly related to the difficulty of placing the transmitter inside the OLTC being diagnosed.

The mounting positions for contact and immersion sensors were determined in preliminary works, where locations of the highest signal to noise ratio (SNR) were selected.

#### 2.1.2. Measurement Settings

The AE signal received was amplified in a 2/4/6 type preamplifier system, which has a possibility of step-by-step amplification adjustment: 20, 40, 60 dB. During measurements in the preamplifier, 20 dB gain was used and, in some cases, the gain value was increased to 40 dB. From the preamplifier, the AE measurement signal was fed to the amplification system, subjecting it to 20 dB of gain. 

The common part of all applied measuring systems is the element of measurement signal acquisition. The time sequences of AE series received were recorded using Acquitek CH 3160 measuring card. Because the measuring card has four measurement channels, the experiments were performed in two series: three sensors were attached in the first one and two in the second one.

The electrical discharges generated during the switching process generate AE signals at frequencies up to 350 kHz. Taking into account the Shannon-Kottlekevich sampling claim, to avoid the effect of mutual overlap of periodically repeated spectra, the sampling frequency should be at least twice as high as the maximum frequency of the analyzed AE signal.

In the measurement practice, a sampling frequency several times higher than that of Nyquist is generally used. In our measurements, the sampling frequency of 1 MHz, with a 12-bit distribution of the AC converter was selected.

In the measurements of AE signals generated by the power switch, 300,000 samples were registered, which allows for the recording of the full switching cycle in 300 ms. Laboratory tests of the selector required increasing the number of samples to 2,000,000 and recording the signals in 2 s. During the OLTC tests in operation, the number of AE signal samples was selected depending on the length of the OLTC drive operation time.

#### 2.1.3. OLTC Defects Considered

As mentioned, for most types of OLTC, the working environment is the insulating oil. During the swiching process, a sound pressure wave is produced, which propagates and reaches the metal tank, where it may be registered by contact transducers. This signal contains information characterizing the operation of the power switch and selector [36]. The result of the operation of oil OLTC is the progressive degradation of its components, including the main contacts of the power switch. Therefore, one of the methods enabling its diagnosis of their mechanical condition is the diagnosis of the degree of contact wear. This most frequent defect was modeled in the research and then the influence of its occurrence on the recorded AE signals was examined. The modeling of the defect consisted of a manual change of the thickness of OLTC-head fixed contacts. This was done successively by: the separation of fixed contacts from the place of their fixing, placing a special pad with a thickness of *d* mm under each of them, and then their reassembly. As a result, the contact working surface was *d* mm closer to the moving main contacts. The specially prepared pads were made of steel sheet. Their shape was selected in such a way as to ensure, on the one hand, good support of the contact and not to allow for assembly clearance, and, on the other hand, not to introduce distortions, curves or other unintentional dislocation of the contact in relation to the original position. After the pads have been placed under each fixed contact, the contacts themselves have been placed in their proper places and then bolted with mounting screws. After the preparation of the model, the functionality of the model was checked to detect potential problems in the operation of the modified device. After a thorough check and confirmation of the correctness of assembly, measurements were taken. The proposed laboratory system allows for the adjustment of contact thickness *d* from 1 to 3 mm, which is related to the actual degree of wear. Moreover, it is possible to simulate the non-uniformity of their operation. Therefore, five classes were considered, marked as:

- Class C1: No damage - power switch operation with new contacts.
- Class C2: Operation of the power switch with 1 mm thick contacts.
- Class C3: Operation of the power switch with 2 mm thick contacts.
- Class C4: Operation of the power switch with 3 mm thick contacts.
- Class C5: Non-simultaneous operation of the power switch contacts.

#### 2.1.4. Measurement Disturbances

When recording AE signals, the possibility of interference must be considered [37,38]. External interference refers to the environmental conditions prevailing in the place of measurement. An example of this type of disturbance, which accompany measurements in real conditions, is the presence of exhaust air discharges occurring in overhead elements of station equipment, electric power lines, culvert isolators, etc. The measuring cable is particularly exposed to electromagnetic interference. Often, due to the location of the tested unit at the substation, the measurements require the use of a cable which average length is several meters. To counteract the influence of electromagnetic interference on the measuring cable, shielding is used. However, a more effective solution is to use a fiber optic link, which ensures the complete elimination of this type of interference. Moreover, it increases the safety of the measurements, as the electrical separation between the measuring transmitter and the rest of the system is applied. 

When analyzing the influence of interferences, it is also important to stress the importance of external electromagnetic type interferences, which include own noise of preamplifiers, amplifiers, and data recording systems [39,40,41,42]. In the case of a small maximum value of the signal measured with the noise of the measuring equipment, there are analytical difficulties related to the extraction of relevant information from the recorded signal. These shots are created on passive and active elements of the measurement track. Among the disturbances generated by the measuring equipment, the following can be distinguished: thermal (thermal), shot (Schottky), flickering, generation-recombination, and explosion noise.

The most important is heat noise (Johnson’s), which is the main source of interference in electronic circuits. They occur on every resistive element regardless of the technology. The mechanism of their formation consists of the interaction of thermal vibrations of the semiconductor’s crystal network with free electrons at temperatures above absolute zero. This movement causes uncontrolled changes in voltage or current values.

The described disturbances especially occur during measurements in real conditions at the substation. During laboratory tests, the influence of interferences is much smaller. Additionally, it should be noted that the interference amplitude is several times lower than the measurement signal generated by the power switch. The described disturbances may be more significant in other types of switches where the amplitude of the measurement signal is not as high as in the examined case. Taking into account the construction of the tested OLTC, the high amplitude of the measurement signal and the measurement conditions, it can be implied that the influence of the described disturbances is negligible.

### 2.2. Data Analysis and Classification Methods

**Figure 1:**
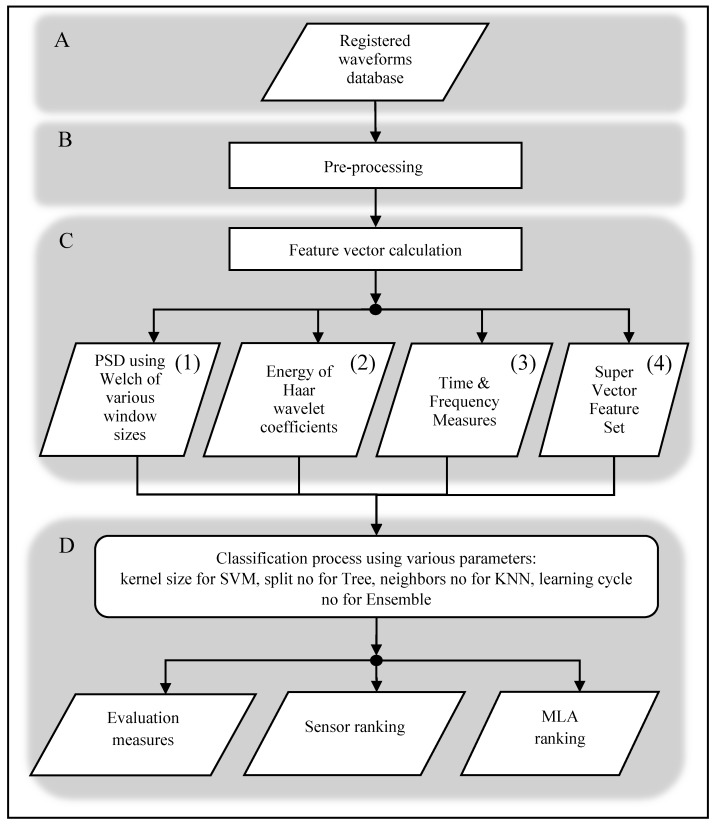
Methodology of the data analysis and classification procedure.

In Figure 1, the methodology of the data analysis and classification procedure is depicted. The procedure is divided into four blocks: A, B, C, D. It starts (A) with gathering signals in a series of measurements. Next, (B) the recorded AE signals are subjected to digital processing analyses, in which the signal is reduced by pre-samples and tail. Similar steps, pre-trigger removing and tail cutting, are taken as a standard procedure, e.g., in [15]. In [15], the authors applied energy criterion for waveform cutting and additionally resampling for lowering the resulted signal. They also proposed an *alter-class matrix* method, which allows for noise and outliers’ introduction. In our case, the signals, registered by various sensors, have different characteristics, and our aim is not to reduce the information included in the registered signals.

In the subsequent step (C), feature extraction takes place. During this phase, a set of features is determined, in particular: the power spectrum density (PSD) using Welch method is calculated for various window sizes, which constituted the (1) *Welch PSD* feature set; based on this, parameters are determined for the *Frequency* feature set, which contains such measures as mean and median frequency, spectra centroid, spectra skewness and spectra kurtosis; furthermore, the energy of detail coefficients, calculated using Haar wavelet decomposition is determined as (2) *Haar Wavelet* feature set. This wavelet method was selected within preliminary works. The base wavelet has a fundamental impact on the results obtained from the discrete waveform transformation, which then affects the calculated energy values of the details. The wavelet selection procedure included calculating the value of the coefficient of variation, which is a classic measure of the variation of results. The procedure was repeated for 29 wavelengths, including Haar, Symlet, and Daubechies of various ranks in the range from 2 to 15. On this basis, the most suitable wavelet was selected: Haar wavelet packet energy percentage, with 6 wavelet packet energy features [43,44,45]. Also in [46], authors analyze AE signals features of which were determined with the use of wavelet transform.

Furthermore, the Hilbert transform was applied to the absolute value of the registered signal amplitude, and the envelope was determined by this means. The envelope was subjected to *Time Feature* set determination, in which the following measures are included: shape and peak coefficients, maximum value, arithmetic, geometric and harmonic mean, median, kurtosis, slope, standard deviation, and variance. The *Time and Frequency Measures* were combined as subsequent (3) features set. Additionally, all the measures were combined into one (4) *Super Vector* features set and applied for classification. Similar methods of feature extraction are performed by other groups of scientists. For example, *Time features and Frequency features* were determined according to reference [47,48] and [15]. In [15], the authors classify AE signals processed with the use of wavelet transformations and using random forest and KNN algorithms to locate corrosion in devices applied in the chemical industry. After the features are determined, they are applied for the (D) classification process, which results in a ranking of the sensors and MLA. This step is described in detail further in this section.

**Figure 2:**
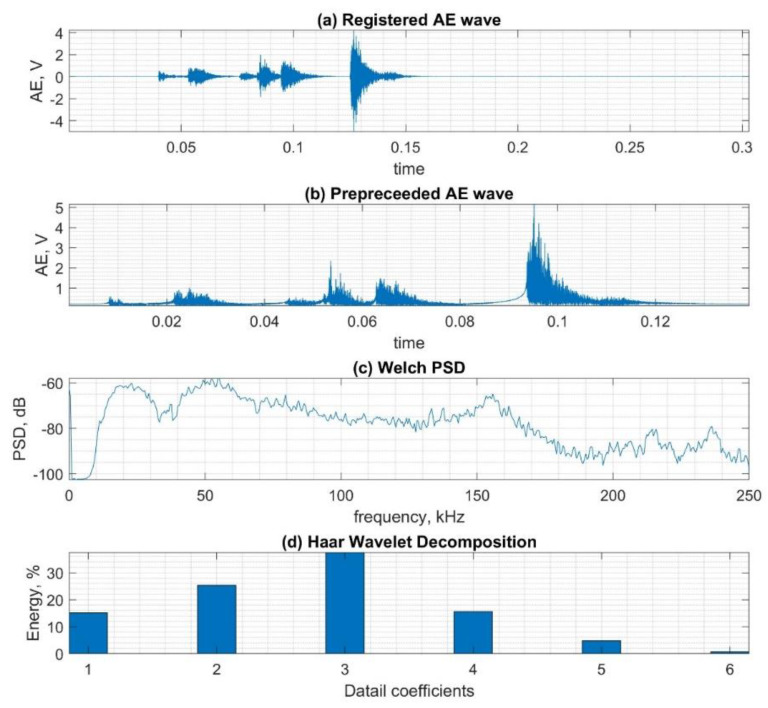
Process of preparing data for classification, considering sample signal for class C1 sensor type R15. (**a**) Registered waveform, (**b**) pre-processed signal, (**c**) power spectrum density calculated using the Welch method, (**d**) Energy of wavelet decomposition coefficients calculated using Haar wavelet.

In Figure 2, a graphical representation of the major methodology steps (see Figure 1B,C) is presented. The examplary AE waveform was registered using the R15 sensor during the simulation of defect D1 (without damage). The original waveform is depicted in Figure 2a. The envelope is depicted in Figure 2b. The PSD Welch signal, calculated for the size of the window of 1024, is depicted in Figure 2c, and the energy for six Haar wavelet coefficients is depicted in Figure 2d.

**Figure 3:**
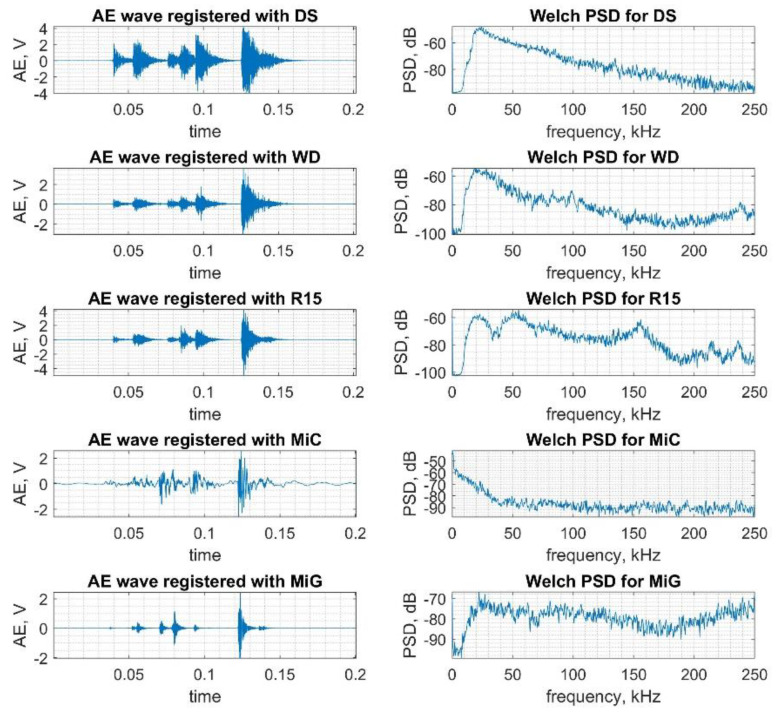
Example waveforms and corresponding Welch power spectrum density (PSD) of class C1 signals registered with the considered sensors.

In Figure 3, the example waveforms and corresponding Welch PSD of class C1 signals registered with all considered sensors are presented. Significant differences in the waveforms and power spectra may be recognized. 

The environment applied for analysis and classification was Matlab. It has a set of built-in artificial intelligence algorithms for classification, including supervised MLA and neural networks. The application of this environment allows one to use the resources of modern computing methods without the need to implement these algorithms from scratch. In this situation, it was possible to test a large group of algorithms, available in this tool, along with the modification of their parameters. Four groups of algorithms were tested: support vector machines (SVM), ensemble, k-nearest-neighbors (KNN), and decision trees. In the group of SVM algorithms, four types of kernel functions were studied: linear, quadratic, cubic, and Gaussian. In the group of KNN algorithms, three types of distance measurement functions (distance metric) were studied: Euclidian, cubic Minkowski, and cosine. In the group of ensemble algorithms, three types of algorithms were tested: bugged tree, subspace discriminant, and subspace KNN. To facilitate subsequent analysis, the following designations have been introduced for the tested MLA: SVL (support vector machine with the linear kernel), SVC (support vector machine with the cubic kernel), SVQ (support vector machine with the quadratic kernel), SVG (support vector machine with Gaussian kernel), ESD (ensemble subspace discriminant), EBT (ensemble bugged tree), ESK (ensemble subspace KNN), KNE (k-nearest-neighbors with Euclidian distance metric), KCO (k-nearest-neighbors with cosine distance metric), TRE (decision tree), and KCM (k-nearest-neighbors with cubic Minkowski distance metric).

The proposed methods of classification resulted from the fact that a relatively small amount of measurement data is available (see Table 1). The signals were gathered under laboratory conditions where the particular defects were modeled; thus, the expert knowledge about the defect was given a priori. Such experiments, using OLTC, are relatively difficult to perform, and the number of units available for measurements is small. Therefore, the application of, e.g., neural networks or deep networks would require “data-augmentation” [6]. Such methods are commonly used in various industry areas, e.g., in processing and classifying images. To ensure a proper learning process of the MLA on a relatively small number of training data, the cross-validation method with a coefficient of 5 was applied during the classification process.

For the above-mentioned procedure, carefully selected signals have been used, i.e., there are no erroneous signals in the learning vector or such with strong interferences. It is important for the supervised learning algorithm not to teach it with erroneous signals or with such low SNR. The interferences and distortions of low amplitude that occur in OLTC are still contained in the AE signals we considered in the classification process. High-quality signals with high SNR value are assumed for the proposed MLA. Application in cases of strong AE interferences can be considered in future works.

In [49], the authors examined the influence of the number of features on the classification results obtained using KNN, RF, and SVM. The evaluation was based on the analysis of accuracy, sensitivity, specificity, precision, and F1 score measures. Their analysis clearly shows that, regardless of the type of algorithm, the values of quality measures increase as the number of features increases, with a maximum of 650 features tested. The highest increases in accuracy were observed for the first few dozen feature elements. For a higher number, these increases were not much larger. In our case, we consider the following sizes of feature sets: *Welch PSD* (129 features), *Haar Wavelet* (6 features), *Time and Frequency Measures* (16 features), and *Super Vector* (151 features). As we will show later in this paper, no significant differences were observed between the *Welch PSD* and *Super Vector* feature sets. 

As a measure of quality, validation accuracy was used in the analysis of results presented in detail in the following section. However, other quality measures were also considered in the analyses: test accuracy, sensitivity, specificity, precision, F1 score, and Matthews correlation coefficient (Matthews CC). Additionally, the CPU time was calculated. The results, included in the next section, allow us to state that, in the considered problem, there exists no CPU time issue, since the CPU times are fractions of seconds, except for a few MLA parameters, for which the analyses have taken a few minutes, but did not affect (improve) the quality of the classification. On this basis, it was possible to identify specific MLA parameters for which the best results were achieved. These results were found in the validation process based on the mentioned quality measures.

In further sections of the article, detailed results for the *Super Vector* feature set, for which the best values of evaluation parameters were obtained, are presented. For the other feature sets, the results depicting the summary statements, from which the ranking can be determined, are shown. 

## 3. Results and Discussion

### 3.1. Analysis of the Influence of MLA Parameters on the Results Obtained for the Super Vector feature Set

To select the optimal values of classification algorithms, an analysis of the influence of particular parameter values on classification efficiency was carried out. The parameters were changed depending on the type of algorithm as follows: SVM—*the size of kernel scale* in the range from 0.1 to 50, decision tree—*no of tree splits* in the range from 1 to 200, KNN—*no of neighbors* in the range from 1 to 100, ensemble—*no of learning cycles* in the range from 1 to 100. The tests were performed for each of the methods separately for signals recorded by individual sensors.

**Figure 4:**
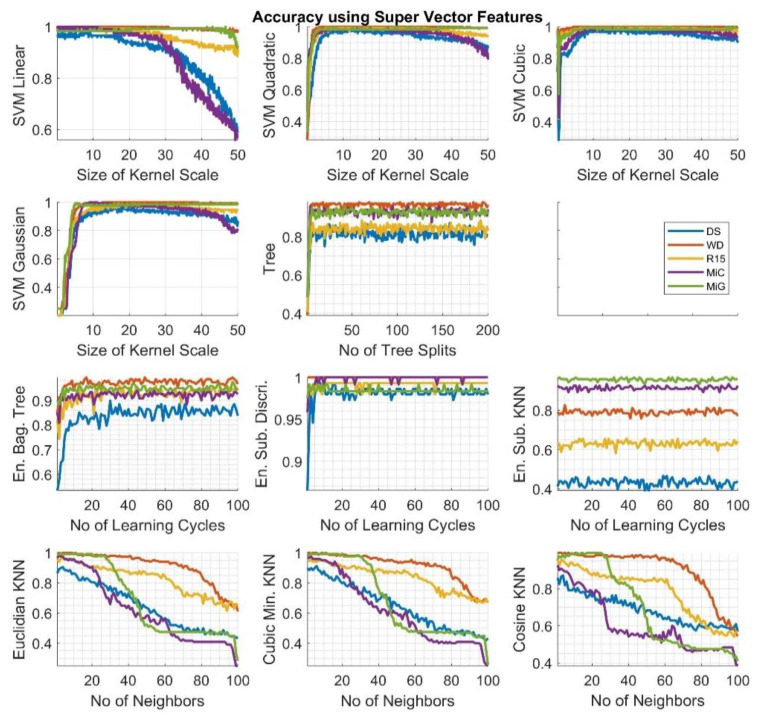
Calculation results—the validation accuracy value for particular algorithms using the *Super Vector* feature set. The legend for the colors corresponding to the individual sensors can be found on the right side of the image.

In Figure 4, the calculation results, which depict the validation accuracy values gathered for particular algorithms using the *Super Vector* features set for the classification process are presented. The colors of particular lines correspond to data related to signals registered by the individual sensors.

Depending on the type of kernel function used for classification and the type of sensor with which AE signals were recorded, differences were observed as follows. 

For algorithms type SVM:

- With a first degree polynomial (SVL), the highest efficiency is achieved for the smallest kernel scale. It then decreases with the increase of the scale size, but not for every type of signal. For signals recorded with DS and MiC sensors it drops rapidly, even below 0.6; for R15 to 0.9; and, for WD, the accuracy is the highest, regardless of the size of the analyzed parameter. In contrast, for MiG sensors, the value is initially smaller, then increases, so that, for values above 40, it falls again, but with values close to 0.9.
- This situation is different for the higher orders of the polynomial (SVQ, SVC) used and with the Gauss function-core (SVG). However, in these cases, the effectiveness is initially worse and increases for increasing kernel sizes.

For decision trees and ensemble algorithms:

- Efficacy is not dependent on the number of tree splits or from the number of learning cycles, especially for the ESK algorithm. However, significant differences in these values are visible depending on the type of sensor used.
- For Tree, EBT, and ESD the values are worse for the smallest value of the parameter and then increase and stabilize at a constant level.
- The worst results were obtained for the ESK algorithm and here also the biggest disproportions are visible for the signals recorded by individual sensors.

For algorithms in the KNN family:

- For each type of applied kernel function, there is a negative impact on the level of effectiveness of the increase in the number of neighbors. Each time, the best results were obtained for the WD and the MiG sensors.

Fluctuations in the accuracy value for the successive values of the parameters under consideration result from the fact that the learning process uses the method of cross-validation, which each time divides the sequence into sets of learning, validation, and test, which causes the results to be not deterministic, but avoids over-learning, which is important in our case because we have only a limited number of measurement samples (see Table 1). 

**Figure 5:**
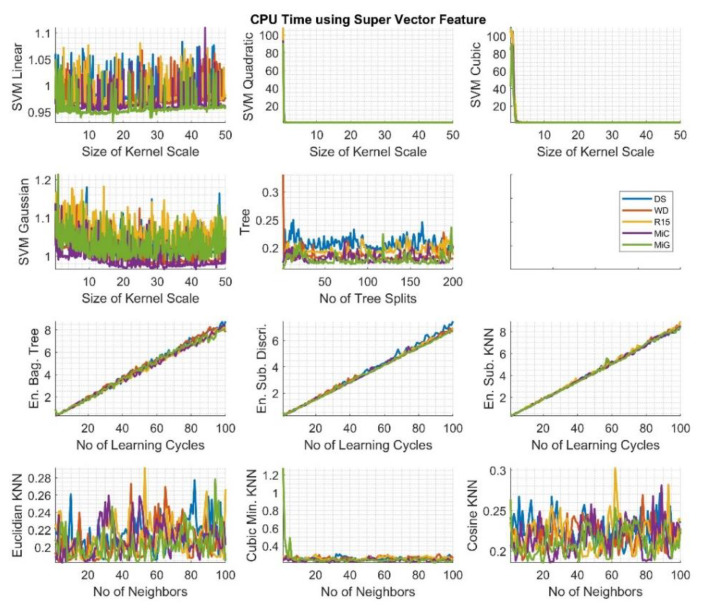
Calculation results—the CPU time for particular algorithms using the *Super Vector* feature set. The legend for the colors corresponding to the individual sensors can be found on the right side of the image.

Graphs in Figure 5 depict the duration of the learning and prediction process (CPU time), which varied depending on the type of algorithm and its parameters used. However, no significant differences in the classification of signals recorded by individual sensors were observed. In the case of Euclidian, cubic Minkowski, and cosine KNN algorithms, these times are maximum one second for the smallest area of analysis (number of neighbors equal to one) and a fraction of a second for the remaining sizes of analyzed areas of adjacent classes.

The situation is similar for decision trees, which, regardless of the number of splits, do not exceed a fraction of a second. In the case of algorithms from the ensemble group, the CPU time increases linearly relating to the increasing number of learning cycles, but, even for 100 iterations, the time is only eight seconds. In the case of algorithms from the SVM group, the duration does not significantly depend on the number of kernel scale sizes, and, excluding the smallest kernel, it is about one second. Only for SVM with the kernel function of the second- and third-order polynomial type, for the smallest size of the kernel scale, the CPU time exceed 100 s. 

**Figure 6:**
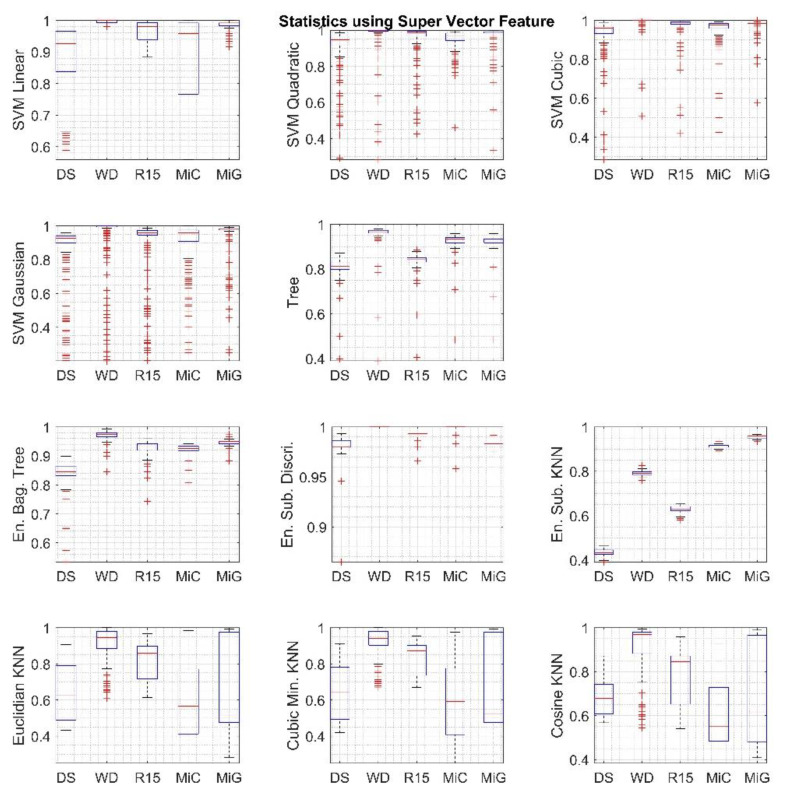
Boxplots calculated over the gathered classification results using the *Super Vector* feature set. Each column in the figures corresponds to a different sensor.

To determine the specific values of parameters, which are the most suitable for the signals, a statistical analysis of the obtained classification results was performed based on validation accuracy measure, the results of which are presented in the form of boxplots in Figure 6. Based on boxplot diagrams, it is very clear that the best outcome is achieved when classifying signals recorded with the WD sensor. However, for other sensors, the situation is no longer obvious and changes depending on the type of algorithm used. The results of this analysis also indicate that different results are obtained depending on the type of MLA algorithm, for example, the smallest deviations and the highest values are obtained for the ESD algorithm. 

The analysis of the data shown in the figures above has allowed for determining the following:

- The optimal kernel scale value for SVM algorithms was selected at level 10.
- The optimal value of tree splits for the decision tree algorithm was chosen at level 20.
- The optimal number of learning cycles for ensemble group algorithms was chosen at level 40.
- The optimal number of neighbors for KNN algorithms is chosen at level 1, which is the most precise.

**Figure 7:**
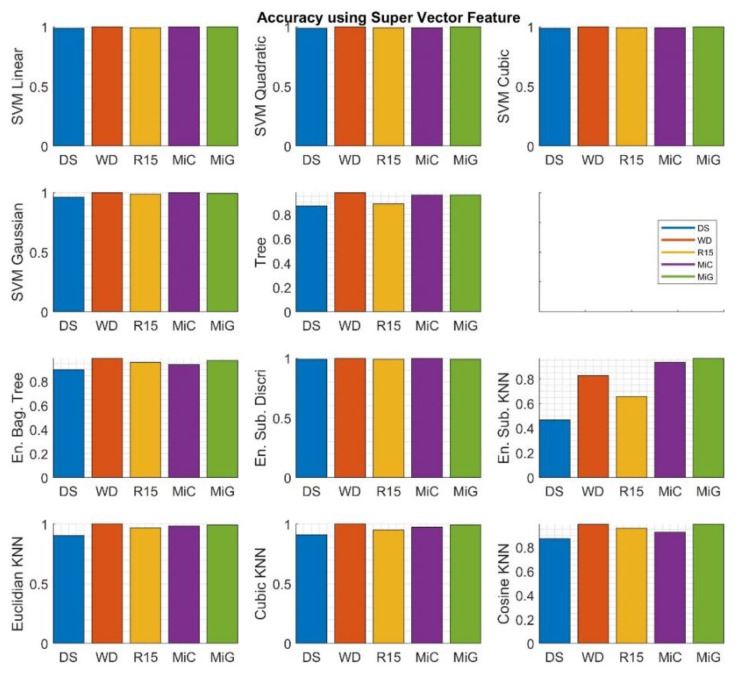
Very best validation accuracy values calculated for each of the algorithms and sensors depicted as a bar plot related to the *Super Vector* feature set.

Figure 7 presents the results of calculations for each highest accuracy value obtained for signals recorded by individual sensors. It can be observed that, in most cases, a good or very good match is obtained. In the case of the ESK, the results were the worst. The presented data are related to the *Super Vector* feature set applied in the classification process.

### 3.2. Discussion on the Optimal Sensor and MLA with Dependence from the Applied Feature Set

**Figure 8:**
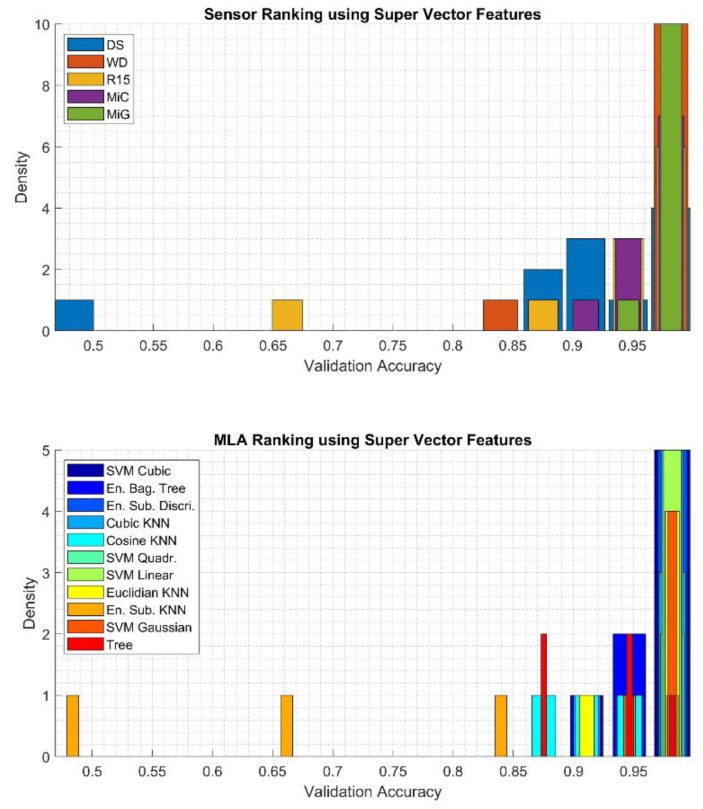
Results of analysis depicting quantities of the best sensor (upper) and the best machine learning algorithm (MLA) (bottom) applied for classification using the *Super Vector* feature determined using validation accuracy value.

**Figure 9:**
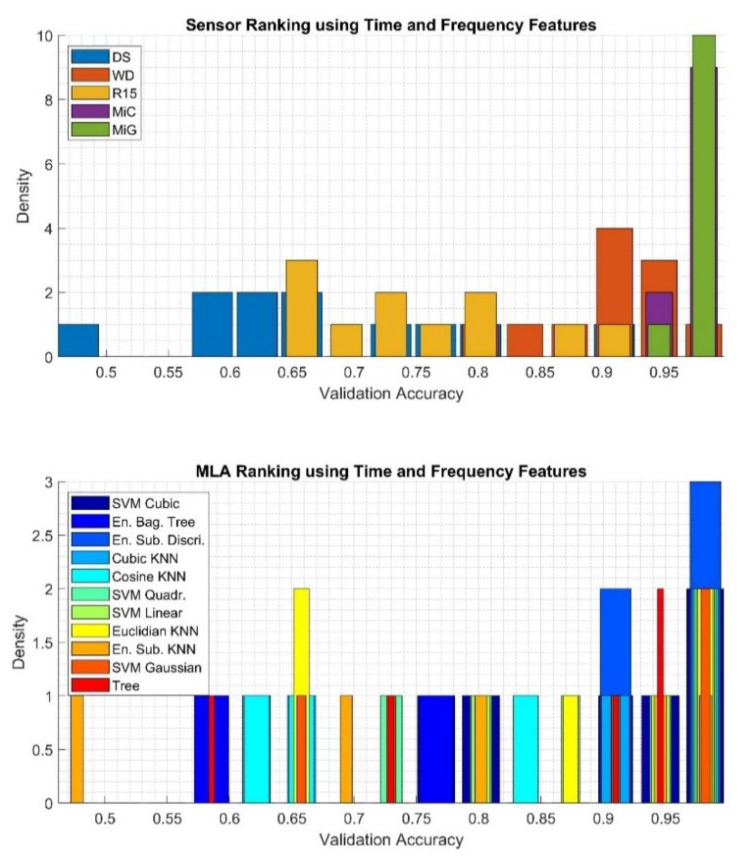
Results of analysis depicting quantities of the best sensor (upper) and the best MLA (bottom) applied for classification using the *Time and Frequency* feature set determined using validation accuracy value.

**Figure 10:**
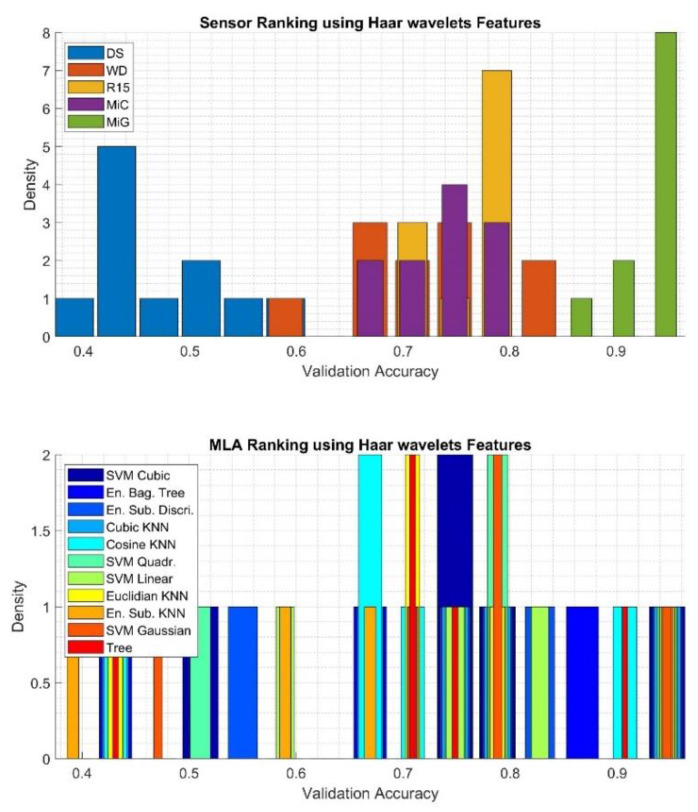
Results of analysis depicting quantities of the best sensor (upper) and the best MLA (bottom) applied for classification using the *Haar wavelets* features determined using validation accuracy value.

**Figure 11:**
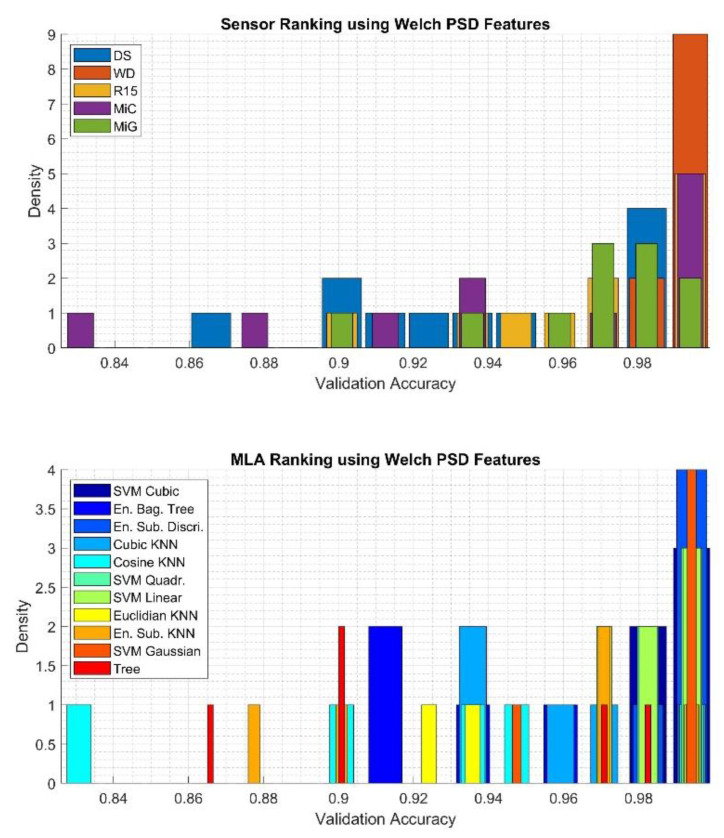
Results of analysis depicting quantities of the best sensor (upper) and the best MLA (bottom) applied for classification using the *Welch PSD* features determined using validation accuracy value.

To qualitatively assess the obtained results, histograms were calculated using 15 bins, based on which it was determined which of the sensors applied for the recording of the AE signals is best suited for classification and which of the MLA most often achieved highest efficiency, regardless of the type of sensor used. The results related to classification using the *Super Vector* feature are presented in Figure 8. The results related to *Time and Frequency* features, *Haar wavelet* features, and *Welch PSD* features are presented in Figure 9, Figure 10 and Figure 11, respectively.

The very best results were obtained in the classification process with the *Super Vector* feature set, which is presented in Figure 8. In this case, the highest validation accuracy values were obtained for signals recorded with the WD and MiG sensors (density equal to 10). Similar results, with density equal to 7 and 6, were obtained for signals recorded by sensors MiC and R15. Values below 0.6 were obtained for the DS sensor. Any type of algorithm from the SVM family, ESD, or KNE can be successfully used for this type of data set due to similar validation accuracy values. It is not recommended to use an ESK algorithm for which the lowest values of the quality measure under consideration were obtained. 

Based on data presented in Figure 9 top, which is related to the results gathered using the *Time and Frequency* feature set, one can recognize that AE signals registered using MiG and MiC sensors achieved, most often, the highest validation accuracies: 10 and 9, respectively. Much worse results were obtained for the classification of signals registered using the DS sensor. While the signals recorded by the R15 sensor were average to good. Based on data presented in Figure 9 bottoms, which depict the results of the evaluation of the applied algorithm, it can be stated that all algorithms achieved similar results. The very best results were gathered for the ESD, while the worst were obtained using the ESK algorithm.

Based on data presented in Figure 10 top, which is related to results gathered using the *Haar wavelets* feature set, it can be seen that by far the best results are achieved for signals registered with the MiG sensor. Moderate validation accuracy values, in the range 0.6-0.8, were gathered for signals registered by WD, R15, and MiC, while the worst were gathered for signals registered by the DS sensor. In terms of determining the optimal algorithm, the evaluation is not trivial because all algorithms obtain flat density distributions. In most cases, the worst results were obtained for the ESK algorithm.

Based on the data presented in Figure 11 top, which is related to the results gathered using the *Welch PSD* feature set, one can recognize that AE signals registered using the WD sensor were most often classified achieving the highest validation accuracy (density equal to 9). Twice as infrequently are such good results obtained for the classification of signals recorded with R15 and MiC sensors, while only in two cases were such good classification results obtained for signals recorded with MiG sensors. Slightly worse results were obtained for the classification of signals with the DS sensor. It should be noted that the worst classification results were obtained in the case of signals recorded with MiC (validation accuracy less 0.84). Based on data presented in Figure 11 bottom, which depicts the results of analysis concerning the evaluation of the applied algorithm and kernel functions, it can be stated that ESD and SVG achieved four times the best results, while SVL, SVC, and SVQ were as good in classification one time fewer. The worst results were obtained using the KCO algorithm.

In Table 2, summarized results gathered from comparative analysis obtained for all four considered feature sets using six various measures of classification quality are depicted. The presented data include the best and the worst cases: the maximal and minimal calculated values of the measure, the algorithm for which it was determined, and the sensor, with which the AE signals were registered. As has already been stated several times in this paper, based on analyses of validation accuracy, here, in an overall comparison of the results obtained, it was confirmed that the highest values were obtained for the *Super Vector* feature set, ESD algorithm, and the WD sensor. 

The same results were obtained for the *Welch PSD* feature set. In contrast, using *Haar wavelets*, the MiG sensor turns out to be the best for measuring AE signals and the SVL algorithm for classification. When using the *Time–Frequency* feature set, the most appropriate sensor is the MiC, while, in the classification task, similar results are obtained for the ESD and SVL algorithms. The analysis of the lowest values led to the unequivocal statement that the worst-fitting algorithm was SVG only for the following parameters: accuracy, sensitivity, specificity, and precision. On the other hand, the F1 score and Matthews correlation coefficient parameters indicate different algorithms (SVQ, SVC, and EBT) depending on the applied feature set as the training sequence. The worst results for the vast majority were obtained for signals recorded with the DS sensor. In individual cases, the signals recorded with WD and R15 sensors were also poorly classified.

## 4. Conclusions

Based on gathered analysis results, and, in particular, the ranking presented in Table 2, it is possible to consider the differences calculated for the individual sensors. Additionally, one can investigate the differences calculated for individual classification methods.

Summarizing the obtained results, it can be stated that each time choosing the optimal set of parameters of the learning algorithm, including the kernel function, it is possible to classify the defect with a very high level of accuracy using the power density spectrum analysis calculated with the Welch method (*Welch PSD)*. Identical results were obtained for the largest set of features under consideration, the *Super Vector* feature set. In both cases, the best results were obtained for AE signals recorded with the WD sensor and applying the ESD MLA. For the other types of analysis, the values of the assessment quality measures exceed 94%. When wavelet analysis is used, the best results were obtained for the MiG sensor. When considering *Time–Frequency Measures*, the MiC sensor is best used. 

The DS has a bandwidth of only up to 180 kHz and this is probably the reason why the worst classification values were obtained for the signals recorded with it. In comparison to this, the WD sensor’s bandwidth is the widest—up to 1MHz. For the R15 sensor, with a bandwidth up to 400 kHz, average results were obtained in comparison with other sensors. In the case of the MiG hydrophone, the band is also up to 180 kHz, and yet, with the use of wavelets, a very good match was achieved, especially for SVL MLA. Moreover, for the MiC sensor, which has a bandwidth of up to 800 kHz, good classification results were obtained using ESD and SVL.

The obtained results may be used in automated expert systems for diagnostic purposes, where the knowledge base will include fingerprints derived from the digital processing of recorded signals. The system may contain different types of MLA algorithms that will recognize the OLTC defect depending on the type of sensor used for measurement.

## Figures and Tables

**Table 1:** Number of samples used for each sensor related to the class considered.

Class no	Number of Samples Used for Each Sensor				
	DS	WD	R15	MiC	MiG
**C1**	29	29	29	15	15
**C2**	30	30	30	27	27
**C3**	30	30	29	19	19
**C4**	30	30	30	30	30
**C5**	29	30	30	29	29

**Table 2:** Summary of the best and worst values of considered measures, depicting the related MLA and sensor, related to the various feature sets.

Measure Name	Super Vector F.	Welch PSD F.	Haar Wavelet F.	Time–Frequency F.
	Best Value of the Measure, MLA, Sensor			
Accuracy Sensitivity	1, ESD, WD 1, ESD, WD	1, ESD, WD 1, ESD, WD	0.958, SVL, MiG	0.991, ESD, MiC
			0.961, SVL, MiG	0.993, ESD, MiC
Specificity	1, ESD, WD	1, ESD, WD	0.989, SVL, MiG	0.998, ESD, MiC
Precision	1, ESD, WD	1, ESD, WD	0.957, SVL, MiG	0.993, SVL, MiC
F1 score	1, ESD, WD	1, ESD, WD	0.958, SVL, MiG	0.992, SVL, MiC
Matthews CC	1, ESD, WD	1, ESD, WD	0.948, SVL, MiG	0.990, SVL, MiC
	**Worst value of the measure, MLA, Sensor**			
Accuracy	0.201, SVG, WD	0.203, SVG, WD	0.202, SVG, DS	0.201, SVG, WD
Sensitivity	0.200, SVG, DS	0.200, SVG, DS	0.200, SVG, DS	0.200, SVG, DS
Specificity	0.800, SVG, DS	0.800, SVG, DS	0.800, SVG, DS	0.800, SVG, DS
Precision	0.286, SVQ, WD	0.298, SVQ, DS	0.258, EBT, DS	0.359, SVC, DS
F1 score	0.252, SVQ, DS	0.286, SVQ, DS	0.254, EBT, DS	0.270, SVC, R15
Matthews CC	0.109, SVQ, DS	0.135, SVQ, DS	0.089, EBT, DS	0.162, SVC, DS
